# Ageing‐associated increase in SGLT2 disrupts mitochondrial/sarcoplasmic reticulum Ca^2+^ homeostasis and promotes cardiac dysfunction

**DOI:** 10.1111/jcmm.15483

**Published:** 2020-07-11

**Authors:** Yusuf Olgar, Erkan Tuncay, Sinan Degirmenci, Deniz Billur, Rimpy Dhingra, Lorrie Kirshenbaum, Belma Turan

**Affiliations:** ^1^ Departments of Biophysics Ankara University Faculty of Medicine Ankara Turkey; ^2^ Departments of Histology‐Embriyology Ankara University Faculty of Medicine Ankara Turkey; ^3^ St. Boniface Hospital Albrechtsen Research Centre Institute of Cardiovascular Sciences University of Manitoba Winnipeg MB Canada

**Keywords:** ageing‐heart, Ca^2+^ homeostasis, cardiovascular function, mitochondria, reactive oxygen species, sarcoplasmic reticulum, sodium/glucose cotransporter 2

## Abstract

The prevalence of death from cardiovascular disease is significantly higher in elderly populations; the underlying factors that contribute to the age‐associated decline in cardiac performance are poorly understood. Herein, we identify the involvement of sodium/glucose co‐transporter gene (SGLT2) in disrupted cellular Ca^2+^‐homeostasis, and mitochondrial dysfunction in age‐associated cardiac dysfunction. In contrast to younger rats (6‐month of age), older rats (24‐month of age) exhibited severe cardiac ultrastructural defects, including deformed, fragmented mitochondria with high electron densities. Cardiomyocytes isolated from aged rats demonstrated increased reactive oxygen species (ROS), loss of mitochondrial membrane potential and altered mitochondrial dynamics, compared with younger controls. Moreover, mitochondrial defects were accompanied by mitochondrial and cytosolic Ca^2+^ ([Ca^2+^]_i_) overload, indicative of disrupted cellular Ca^2+^‐homeostasis. Interestingly, increased [Ca^2+^]_i_ coincided with decreased phosphorylation of phospholamban (PLB) and contractility. Aged‐cardiomyocytes also displayed high Na^+^/Ca^2+^‐exchanger (NCX) activity and blood glucose levels compared with young‐controls. Interestingly, the protein level of SGLT2 was dramatically increased in the aged cardiomyocytes. Moreover, SGLT2 inhibition was sufficient to restore age‐associated defects in [Ca^2+^]_i_‐homeostasis, PLB phosphorylation, NCX activity and mitochondrial Ca^2+^‐loading. Hence, the present data suggest that deregulated SGLT2 during ageing disrupts mitochondrial function and cardiac contractility through a mechanism that impinges upon [Ca^2+^]_i_‐homeostasis. Our studies support the notion that interventions that modulate SGLT2‐activity can provide benefits in maintaining [Ca^2+^]_i_ and cardiac function with advanced age.

## 1 INTRODUCTION

Over the past several decades, the average lifespan of humans has increased worldwide. Notably, the prevalence of death from cardiovascular disease is significantly higher in elderly populations.^1^ The epidemiological and demographic results show the development of new therapies and interventions to mitigate the ageing‐related decline in cardiac performance. Growing evidence suggests that ageing triggers cellular defects at the molecular and genetic levels which can result in organ dysfunction.^2^ In this regard, both sarcoplasmic reticulum (SR) and mitochondrial dysfunctions have been linked to ageing‐associated disorders including cardiac dysfunction and contractile failure.^3^

Mitochondrial function is essential for oxidative metabolism and ATP synthesis for maintaining tissue homeostasis and cell survival. Also, the mitochondrion serves as a platform for calcium regulation and programmed cell death by apoptosis and necrosis.^4^, ^5^ Hence, the loss of normal mitochondrial function commonly associated with advanced age has been linked to abnormalities in sarcolemmal Ca^2+^ transport and alteration in action potential duration.^3^, ^6^, ^7^ However, the relationship between mitochondrial calcium handling defects and contractile dysfunction associated with ageing remains poorly understood.

Notably, we recently demonstrated that sodium/glucose co‐transporter 2 (SGLT2), which is a member of the solute carrier family (SLC5A2) and is important in facilitating sodium‐dependent glucose‐transport and in the regulation of blood glucose levels, was significantly activated in the hearts of insulin‐resistant animals with metabolic syndrome (MetS). Furthermore, we demonstrated that pharmacological inhibition of SGLT2 improved glucose tolerance and cardiac function.^8^ SGLT2 inhibitors have also been shown to provide clinical benefits in normalizing glucose levels and cardiac function in diabetic and non‐diabetics patients with insulin resistance.^9^, ^10^ While the underlying mechanisms by which SGLT2 inhibition confers cardioprotection are not well understood, several theories have been proposed including improved haemodynamics, more efficient metabolic function and cardiac/renal effects among others. In particular, a recent study by Pasternak et al^10^ demonstrated in the Scandinavian Cohort study that SGLT2 inhibitors significantly reduced the risk of heart failure and all‐cause mortality, particularly in elderly patients. This finding raised awareness that SGLT2 inhibitors may play an important role in improving cardiac performance in the aged population. This view was substantiated by another recent study by Cintra et al,^11^ who also showed beneficial effects of SGLT2 inhibition in elderly patients with heart diseases. At present, there are several theories that the underlying cardioprotection conferred by SGLT2 inhibition may converge on common signalling pathways that influence cardio‐metabolic and bioenergetics involving Na^+^/H^+^ exchange‐1, (NHE‐1).^12^, ^13^, ^14^ Indeed, deregulated Na^+^ and Ca^2+^ levels are early drivers of mitochondrial dysfunction, ROS production and cardiovascular morbidity and mortality.^15^ It has been shown that the increased intracellular Na^+^ is an underlying cause of cardiac injury and metabolic dysfunction in diabetic cardiomyopathy, presumably from elevated SGLT2 activity.^14^, ^16^ In this regard, the studies have demonstrated that SGLT2 inhibition suppressed elevated NHE‐1 activity and intracellular Na^+^ levels in cardiomyocytes of diabetic animals.^16^, ^17^ Despite our previous published work and clinical studies linking SGLT2 in the pathogenesis of cardiac dysfunction and heart failure, the underlying mechanism by which SGLT2 inhibition improves cardiac function in the aged myocardium remains poorly understood. Therefore, in the present study, we explored the mechanism by which SGLT2 disrupts cardiac function associated with advanced age. Given that the mitochondria are a major nodal point in the regulation of cardiac metabolism and intracellular Ca^2+^ ([Ca^2+^]_i_), we explored the impact of SGLT2 on mitochondrial Ca^2+^ homeostasis in young and aged cardiac myocytes. Our data provide new important information that SGLT2 disrupts mitochondrial/SR Ca^2+^‐homeostasis and thereby promotes cardiac dysfunction by impairing mitochondrial energy metabolism.

## 2 MATERIALS AND METHODS

### 2.1 Experimental animals

Our experimental protocols and all animal procedures are in coincidence with the guidelines of the Directive 2010/63/EU of the European Parliament on the protection of animals used for scientific purposes and are approved by Ankara University (reference # 2016‐18‐165).

Wistar male rats were grouped as 24‐month‐old (Aged group) vs. 6‐month‐old (Young group) and parameters such as blood glucose levels were analysed as described previously.^8^ Hearts were rapidly excised following anaesthesia with sodium pentobarbital (30 mg/kg bodyweight).

### 2.2 Cardiomyocytes isolation

Cardiomyocytes isolation was performed from the left ventricles of hearts, as described previously.^8^ Briefly, hearts were cannulated in a Langendorff‐perfusion system, followed by digestion with 1‐mg/mL collagenase (Type IV, Worthington, USA) for 30‐35 minutes. Cardiomyocytes were treated with either 100 nmol/L SGLT2 inhibitor (SGLT2i, dapagliflozin, D185360; for 4‐5 hours at 37°C) or 0.1 µmol/L MitoTEMPO for further experiments.

### 2.3 Measurement of mitochondrial superoxide production and Ca^2+^ level by flow cytometry

To determine the mitochondrial superoxide level ([SOX]_Mit_), isolated cardiomyocytes were incubated with 2.5‐µmol/L MitoSOX Red for 30‐minutes at room temperature and cells were washed twice in PBS. Fluorescent intensity was measured by flow cytometry, and data were plotted as dot plots.^18^

The mitochondrial Ca^2+^ level ([Ca^2+^]_Mit_) was measured by incubating cardiomyocytes with 4‐µmol/L Fluo4‐AM Ca^2+^ dye for 45‐minutes at room temperature in extracellular solution contains (in mmol/L) 156 NaCl, 3 KCl, 2 MgSO_4_, 1.25 KH_2_PO_4_, 10 D‐glucose, 2 CaCl_2_ and 10 HEPES, at pH 7.35. Cardiomyocytes are washed with PBS twice and incubated with intracellular solution contains (in mmol/L); NaCl 6, KCl 130, MgCl_2_ 7.8, KH_2_PO_4_ 1, CaCl_2_ 0.4, EGTA 2, EDTA 10, malate 2, glutamate 2, ADP 2 and HEPES 20, at pH 7.1 solution including digitonin (25 mg/mL) and thapsigargin (10‐µmol/L) for 10‐minutes at room temperature. Real‐time measurement of the fluorescence emission from each sample is reported as dot plots measuring the percentage of stained cells with Fluo‐4AM fluorescence.^19^

To validate the mitochondrial membrane integrity under control during measurements, we directly measured the mitochondria Ca^2+^ level but not in the mitochondrial intermembrane space, all experiments were performed in cells under same experimental conditions (using Fluo‐4AM and TMRM loaded permeabilized cells, by flow cytometry) through the determination of mitochondrial Ca^2+^ level and mitochondrial membrane potential (MMP), simultaneously by using confocal microscopy (see Appendix S1 and Figure S1).

### 2.4 Measurement of mitochondrial membrane potential

The mitochondrial membrane potential in cardiomyocytes was measured with a fluorescence‐based assay as described previously.^20^ Briefly, a membrane‐permeant single wavelength fluorescence dye JC‐1 (5‐μmol/L, 30‐minutes) loaded cells were imaged with a confocal fluorescence microscope (Leica TCS SP5). Carbonyl cyanide 4‐(trifluoromethoxy) phenylhydrazone (FCCP; 5‐μmol/L) was used as a positive control for mitochondrial depolarization (MMP).

### 2.5 Measurements of the resting levels of intracellular ions and global transient intracellular Ca^2+^‐changes

The basal levels of intracellular free Ca^2+^, Na^+^ and H^+^ (or intracellular pH) levels ([Ca^2+^]_i_, [Na^+^]_i_, [H^+^]_i_) were measured in resting cardiomyocytes loaded with ion‐specific fluorescence dyes (4‐µmol/L Fura‐2AM for Ca^2+^, 10‐µmol/L SBFI for Na^+^ and 5‐µmol/L SNARF‐1 for H^+^). Fluorescence values were recorded by the use of a ratio‐metric micro‐spectrofluorometer (PTI Ratio master and FELIX software; Photon Technology International, Inc, NJ USA) for Ca^2+^ or a laser scanning microscope (confocal microscopy, Leica TCS SP5, Germany) for Na^+^, as described previously.^8^

To determine the intracellular transient Ca^2+^‐changes (Ca^2+^‐transients) in cardiomyocytes (loaded with Fura‐2AM, 4‐µmol/L), an electric‐field stimulation (with a 10‐ms duration electrical pulses at a frequency of 0.2 Hz) was applied. We took into consideration the potential influences of a temperature‐dependent effect on lowered amplitude of intracellular Ca^2+^‐changes without changes in the time course of measurements,^21^, ^22^ as well as the potential effects of temperature on Ca^2+^‐sensitive fluorescent probes,^23^ we used room temperature for both cell‐loading and recordings from loaded cells. Therefore, fluorescence intensity changes were measured at room temperature by using the micro‐spectrofluorometer (PTI, Lawrenceville, NJ, USA) and FELIX software, as described, previously.^8^

### 2.6 Patch‐clamp experiments

Electrophysiological parameters of cardiomyocytes were recorded by using the Axoclamp patch‐clamp amplifier (Axopatch 200B amplifier, Axon Instruments, USA) at room temperature (23 ± 2°C). Cardiomyocytes were sampled and digitized at 5 kHz using an analog‐to‐digital converter and a software (Digidata 1200A and pCLAMP 10.0; Axon Instruments, USA). The amplifier has been used for either voltage‐clamp mode (voltage‐dependent K^+^‐channel currents, I_K_) or current‐clamp mode (action potentials) to measure either ionic fluxes or membrane voltage changes, respectively, as described previously.^8^

Action potential duration from the repolarization phase at 25, 50, 75, 90% (APD_25_, APD_50_, APD_75_, APD_90_), the resting membrane potential, and the maximum amplitude of action potentials were calculated from original records (at least 15‐20 records/cell).

The Na^+^/K^+^‐pump (NaK) current (I_NaK_) was measured in freshly isolated left ventricular cardiomyocytes, as described previously.^24^ Briefly, NaK inhibition was achieved by 20‐minutes perfusion with 0‐K^+^ solution: NaCl 136, BaCl_2_ 2, MgCl_2_ 1, glucose 10, HEPES 5, NiCl_2_ 5 ve TrisCl 4 (mmol/L) (at pH = 7.4). To activate NaK, cardiomyocytes were reperfused with normal 4 K^+^ solution: (NaCl 140, KCl 4, CaCl_2_ 2, MgCl_2_ 1, glucose10 and HEPES, at pH = 7.4). The holding potential was −30 mV during the experiment.

The Na^+^/Ca^2+^‐exchanger (NCX) current was measured as described elsewhere.^25^ The total NCX current (both inward and outward), I_NCX_, was obtained from protocol composed of descending ramp from +80 to −120 mV at 0.1 mV/ms while holding potential was −40 mV. The external solution consisted of NaCl 130, TEA‐Cl 10, Na‐Hepes 11.8, MgCl_2_ 0.5, CaCl_2_ 1.8, ryanodine 0.005, nifedipine 0.02, glucose 10; pH 7.4 and the pipette solution CsCl 65, CaCl_2_ 10.92, EGTA 20, HEPES 10, MgATP 5, MgCl_2_ 0.5, TEA‐Cl 20 and at pH 7.2.

### 2.7 Measurement of cellular ATP level

The cellular level of ATP in isolated left ventricular cardiomyocytes was measured using a colorimetric ATP assay kit (Abcam, ab83355) with some modification, as described previously.^8^

### 2.8 Histological analysis of cardiomyocytes

Electron microscopy was performed in cardiomyocytes, as described previously.^20^ Ultra‐thin sections were stained with uranyl acetate and lead citrate and cells were observed using an LEO 906 E TEM (80 kV, Oberkochen, Germany). A Sharpeye CCD and Image SP (Germany) digital imaging system were used to photograph.

To quantify the results, the mitochondrial aspect ratio as the ratio of length to width was calculated by using Image SP, the randomly selected five longitudinally arranged micrographs, as described previously.^26^ For each cell, the lengths of 500 to 600 interfibrillar mitochondria were assessed, as described previously.^27^

### 2.9 Western‐blot analysis

The protein samples were prepared from the cells and tissues as previously described.^20^ Equal amount of cardiac cell lysates were run on SDS‐polyacrylamide gels and incubated with antibodies against proteins Mfn1 (sc‐166644, 1:1000), Mfn2 (sc‐100560, 1:1000), Drp1 (sc‐271583, 1:500), SERCA2 (SC‐376235, 1:500), NCX (Santa Cruz, sc‐12972), phospho‐phospholamban‐Ser^16^ (pPLB; sc‐17024, 1:10 000), phospholamban (PLB; sc30142, 1:1000), SGLT2 (ab37296,1:1000). β‐actin (Santa Cruz, sc‐47778, 1:5000) and GAPDH (Cell Signaling, D16H11, 1/5000) were used as housekeeping proteins to detect equal protein loading. Immunoreactive bands were detected by a chemiluminescent reaction (ECL kit, Amersham Pharmacia, USA). The densities of the bands are analysed using ImageJ software, and the results were presented as fold changes.

### 2.10 QRT‐PCR analysis

The mRNA levels were measured as described previously^1^. The fold changes in the genes were analysed based on the comparative (2^−ΔΔCt^) method. Primer sequence for SGLT2 is TCATTGCCGCGTATTTCCTG (forward) and AACACCACAAAGAGCGCATT (reverse).

### 2.11 Reagents and statistical analysis

Chemicals were obtained from Sigma‐Aldrich (St. Louis, MO) unless otherwise stated. Data were collected from different myocyte isolations. Data are presented as Mean ± SEM of at least three independent observations for Western blotting with GraphPad Prism 6.0 (GraphPad software, Inc, La Jolla, CA). To provide better presentation and distribution of Western blot data in terms of median and interquartile range, we used a box and whisker graph. Comparisons between quantitative variables were assessed by the unpaired two‐sided Student's t test at the *P* < 0.05 significance level.

## 3 RESULTS

### 3.1 Altered myocardial ultrastructure and cardiac function in aged hearts

**FIGURE 1:**
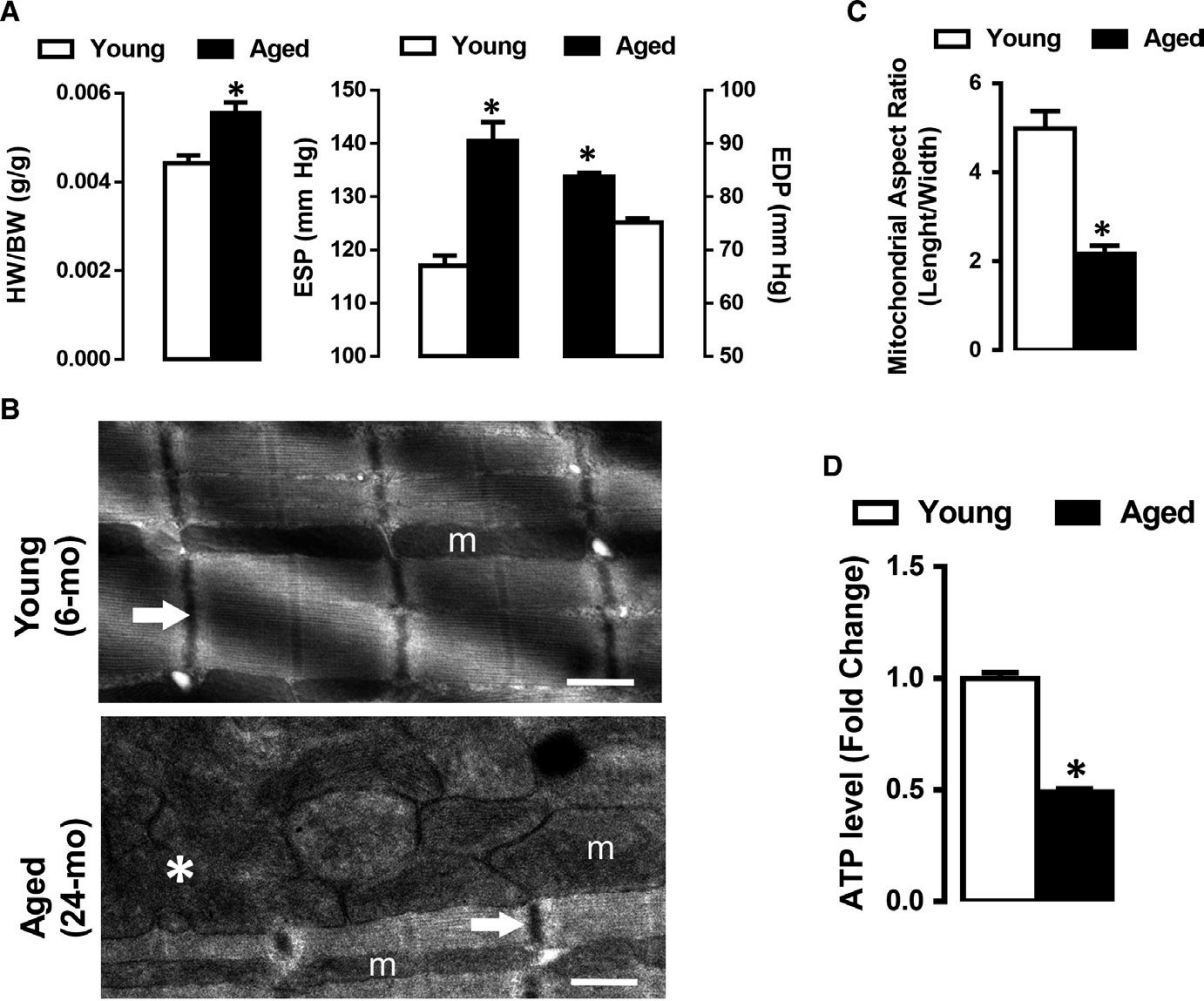
Altered myocardial ultrastructure and defective mitochondria in aged rats hearts. Panel A (Left): The ratio of heart weight to bodyweight in the young group (6 mo old, n = 30) and aged group (24 mo old, n = 30 rats). (Right): End‐systolic (ESP) and end‐diastolic blood pressure (EDP) in young and aged groups. Panel B: Representative transmission electron micrographs (EM) of ventricular cardiomyocytes from young (Top) and aged (Bottom) rat's heart. Normal myocardial ultrastructure with organized mitochondria in young group vs disrupted sarcomeres, unorganized and fragmented mitochondria in the aged group (magnification ×7750). The symbols demonstrate; m for mitochondria, the arrow for Z‐line, asterisk for fragmented mitochondrion. Panel C: Histogram depicts mitochondrial aspect ratio (length/width), from the data derived from EM shown in panel B. (Note: low mitochondrial aspect values are indicative of smaller and fragmented mitochondria). Panel D: Histogram presents fold change in ATP levels (young vs aged group), measured by colorimetric assay. Data are presented as Mean ± SEM, statistical significance ^*^ *P* < 0.05 vs. young group

To gain insight into the effects of ageing on cardiac dysfunction, we first determined whether the aged hearts (24 months) are hypertrophic and different from younger animals (6 months). As shown in Figure 1 panel A (Left panel), the heart weight to bodyweight ratio was significantly higher in the aged group compared with the corresponding young group. Further, end‐systolic pressure (ESP) and end‐diastolic blood pressure level (EDP) were higher in the aged group compared with the younger animal group, Figure 1 panel A (Right panel).

Also, we examined cardiac morphology by electron microscopy to examine ultrastructure and subcellular changes in the myocardium with ageing. Electron microscopy analysis revealed that in contrast to young animals, which displayed normal sarcomeric architecture and organized elongated mitochondria, severe ultrastructural defects characterized by damaged myofibrils, disorganized and fragmented mitochondria accompanied by a dilatation of SR T‐tubules were observed in the aged hearts, Figure 1 panel B.

Further, a significant decrease in mitochondrial aspect ratio was observed in the aged hearts, indicative of increased mitochondrial fragmentation, Figure 1 panel C. Moreover, altered mitochondrial morphology coincides with a marked decline in ATP levels, Figure 1 panel D.

### 3.2 Age‐associated increase in reactive oxygen species (ROS) underlies mitochondrial defects

**FIGURE 2:**
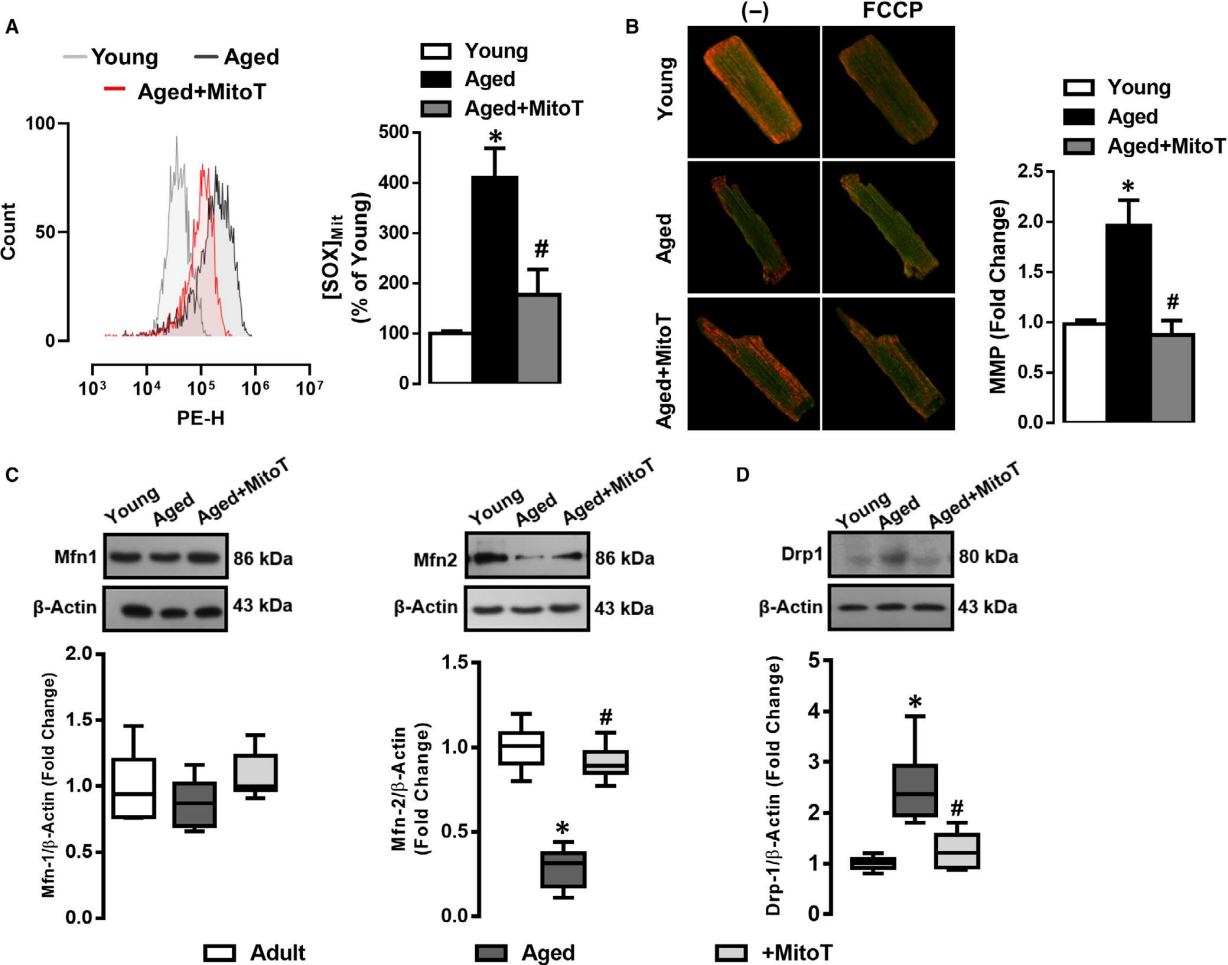
Increased mitochondrial reactive oxygen species (ROS) underlies the loss of mitochondrial membrane potential and altered dynamics in aged cardiomyocytes. Panel A (Left panel): Representative flow cytometric images (left) depicting mitochondrial superoxide [SOX]_Mit_ production in cardiomyocytes loaded with MitoSOX (2.5‐µmol/L for 30‐min at 37°C) for the groups (young, aged and aged + MitoTEMPO), as shown in the labels. Note: MitoTEMPO is a mitochondria‐targeting antioxidant,^20^ treated at 0.1‐µmol/L for 4‐5 h at 37°C. Right panel: Histogram presenting per cent (%) change in values of [SOX]_Mit_; Data are normalized to young controls. Panel B (Left panel): Representative fluorescent images for mitochondrial membrane potential (MMP) in JC‐1 loaded cardiomyocytes, Right panel: Histogram presents fold change in MMP (young vs aged group). Note: Green colour marks loss of mitochondrial membrane potential. Data are presented as Mean ± SEM, statistical significance ^*^ *P* < 0.05 vs. young group, ^#^ *P* < 0.05 vs. aged group. Panel C (Top panel): Western blot analysis of mitochondrial fusion proteins (Mfn1 and Mfn2); Bottom panel: Histogram presents fold change in Mfn1 and Mfn2 proteins. Panel D (Top panel): Western blot analysis for mitochondrial fission protein, Drp1 (D), Bottom: Histogram presents fold change in Drp1 protein. Actin serves as a housekeeping gene. All data are presented, as Mean ± SEM. Cardiomyocytes were isolated from 5‐6 rats/groups. Statistical significance *P* < 0.05 vs. young group, ^#^ *P* < 0.05 vs. aged group, statistical significance analysed by unpaired two‐sided Student's t test

As we observed a decline in mitochondrial ATP in aged animals, suggesting that mitochondrial perturbations may be associated with contractile dysfunction. We used mitochondria targeting antioxidant (MitoTEMPO) that we have previously shown to reduce ROS level in the ventricular cardiomyocytes,^20^ we assessed the effects of ageing on mitochondrial superoxide production in MitoSOX‐loaded cardiomyocytes followed by flow cytometry analysis. As shown in Figure 2 panel A, the fluorescence intensity of MitoSOX was fourfold higher in the aged cardiomyocytes compared with cardiomyocytes from young hearts. Moreover, antioxidant MitoTEMPO exerted a protective effect against ageing‐induced mitochondrial superoxide production, Figure 2 panel A.

As our previous studies demonstrated a role for mitochondrial redox signalling (via increases in ROS level) in the aetiology of ageing‐associated organ dysfunction,^28^ we determined the effect of MitoTEMPO treatment on mitochondrial membrane potential (MMP) in isolated JC‐1‐loaded cardiomyocytes (Figure 2 panel B, original records in the left). The mitochondria were significantly depolarized in the aged cardiomyocytes, which was rescued by MitoTEMPO; FCCP was used as a positive control in the study, Figure 2 panel B.

Taking into consideration the essential processes of mitochondria fidelity such as mitochondrial dynamism (fusion and fission), we determined the expression levels of the mitochondrial fusion and fission proteins in the aged cardiomyocytes compared with young hearts in the absence and presence of MitoTEMPO. As shown by Western blot analysis in Figure 2 panel C, in contrast to Mitofusin 1 (Mfn1), Mitofusin 2 (Mfn2) expression was significantly decreased in cardiac myocytes of aged hearts. Furthermore, a marked increase in mitochondrial fission protein Drp1 was observed in aged cardiomyocytes, Figure 2 panel D. These data are consistent with increased mitochondrial fragmentation and reduced mitochondrial aspect ratio in the aged cardiomyocytes. Interestingly, MitoTEMPO treatment suppressed age‐associated alterations in Mfn2 and Drp1 (Figure 2 panels C,D), suggesting that increased ROS production underlies mitochondrial perturbations and fragmentation associated with the aged hearts.

### 3.3 Effects of ageing on intracellular Ca^2+^ signalling in ventricular cardiomyocytes

**FIGURE 3:**
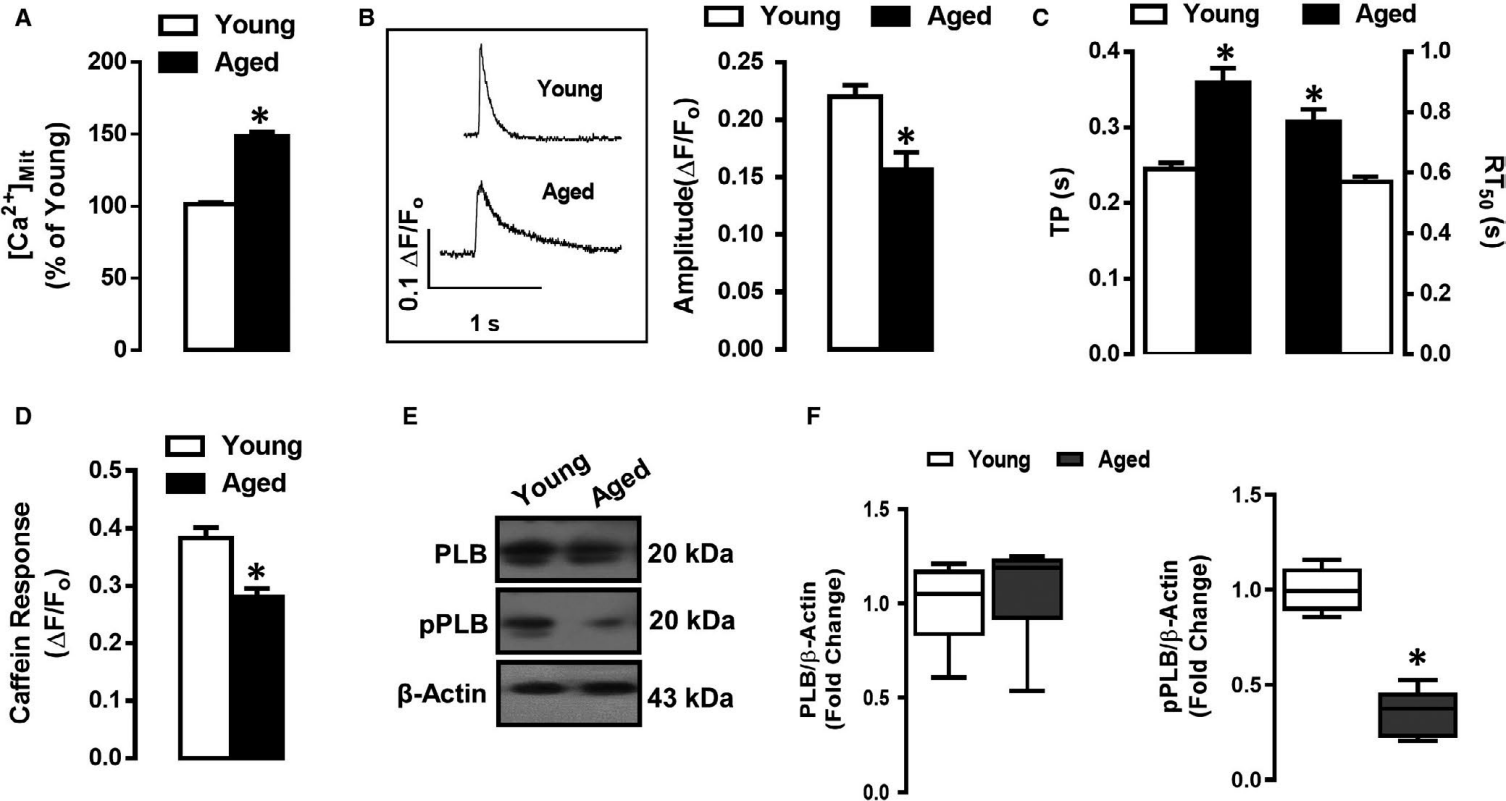
Ageing induced mitochondrial Ca^2+^ overload is coupled with defects in SR Ca^2+^ homeostasis and loss of phosphorylation of phospholamban (pPLB) protein. Panel A, Histogram presenting mitochondrial Ca^2+^ ([Ca^2+^]_Mit_) in the young and old group, measured in 4‐µmol/L Fluo4‐AM Ca^2+^ dye loaded (45‐min) left ventricular cardiomyocytes. Panel B, Representative transient [Ca^2+^]_i_ changes under electrical stimulation (Left panel) and the amplitude of transient [Ca^2+^]_i_ changes (Right panel). Panel C, Corresponding kinetics of transient [Ca^2+^]_i_ changes (TP: the time to the maximum amplitude of [Ca^2+^]_i_ and RT_50_: the half time of re‐uptake of [Ca^2+^]_i_) in aged cardiomyocytes compared with those of young's. Panel D, Assessment of sarcoplasmic reticulum (SR) Ca^2+^‐content obtained following 10‐mmol/L caffeine applications. Panel E, Western blot analysis for total PLB and pPLB levels; Actin served as the equal loading control. Panel F, Histogram presents fold‐change in total PLB and pPLB levels. Data are derived from cardiomyocytes isolated from 5‐6 rats/groups. Data presents the Mean ± SEM values of the groups. Statistical significance *P < 0.05 vs. young group, analysed by unpaired two‐sided Student's *t* test

Elevated mitochondrial Ca^2+^ level ([Ca^2+^]_Mit_) has been identified as a major activator of mitochondrial permeability transition pore (mPTP) opening.^29^ Among other potential influences of mPTP opening, cellular [Ca^2+^]_i_ is considered to be a major determinant of mPTP opening. As ageing alters [Ca^2+^]_i_ handling in cardiomyocytes,^30^ we determined whether mitochondrial Ca^2+^, [Ca^2+^]_Mit_ is elevated in the aged cardiomyocytes. As shown in Figure 3 panel A, (by flow cytometry), [Ca^2+^]_Mit_ was increased by 50% in aged cardiomyocytes compared with those of young cardiomyocytes. To verify the detrimental effect of ageing on depressed contractile activity, we mimicked the action potentials (APs) of age with electrical‐field stimulation of cardiomyocytes and determined the transient changes of [Ca^2+^]_i_ (global [Ca^2+^]_i_ transients) in Fura‐2AM loaded aged cardiomyocytes. As shown in Figure 3 panel B, the averaged peak amplitude (as ΔF_340/380_) of [Ca^2+^]_i_ transients were significantly smaller in the aged group compared with the younger group (Figure 3 panel B). Besides, the time to peak amplitude and the half time for recovery of Ca^2+^ transients in the aged group was significantly longer than those of the young group (Figure 3 panel C).

To test whether the [Ca^2+^] content of the sarcoplasmic reticulum (SR) could be altered with ageing, we performed further experiments using caffeine to assess SR Ca^2+^ extrusion. The responses to acute caffeine (10‐mmol/L) were significantly lower in the aged group compared with the corresponding young group (Figure 3 panel D). To characterize the underlying mechanisms for altered Ca^2+^ transients, we monitored the protein expression of total and phosphorylated phospholamban (pPLB), which modulates the SR Ca^2+^‐ATPase (SERCA) function. As shown in Figure 3 panels E,F, a marked decrease in phosphorylation of PLB (pPLB) was observed in aged cardiomyocytes.

### 3.4 Ageing up‐regulates SGLT2 and disrupts the intracellular ionic balance of cardiomyocytes

As action potential durations were prolonged in aged cardiomyocytes, we assessed whether intracellular ionic levels were altered in aged cardiomyocytes. To test this possibility, we assessed changes in the Na^+^‐influx (via voltage‐dependent Na^+^ channel current, I_Na_) and Ca^2+^‐influx (via voltage‐dependent L‐type Ca^2+^‐channel currents, I_CaL_) on the AP characteristics. However, there was no significant change in any parameter of the channel currents between young and aged groups (Figure S1 and Figure S2, panels A,B).

**FIGURE 4:**
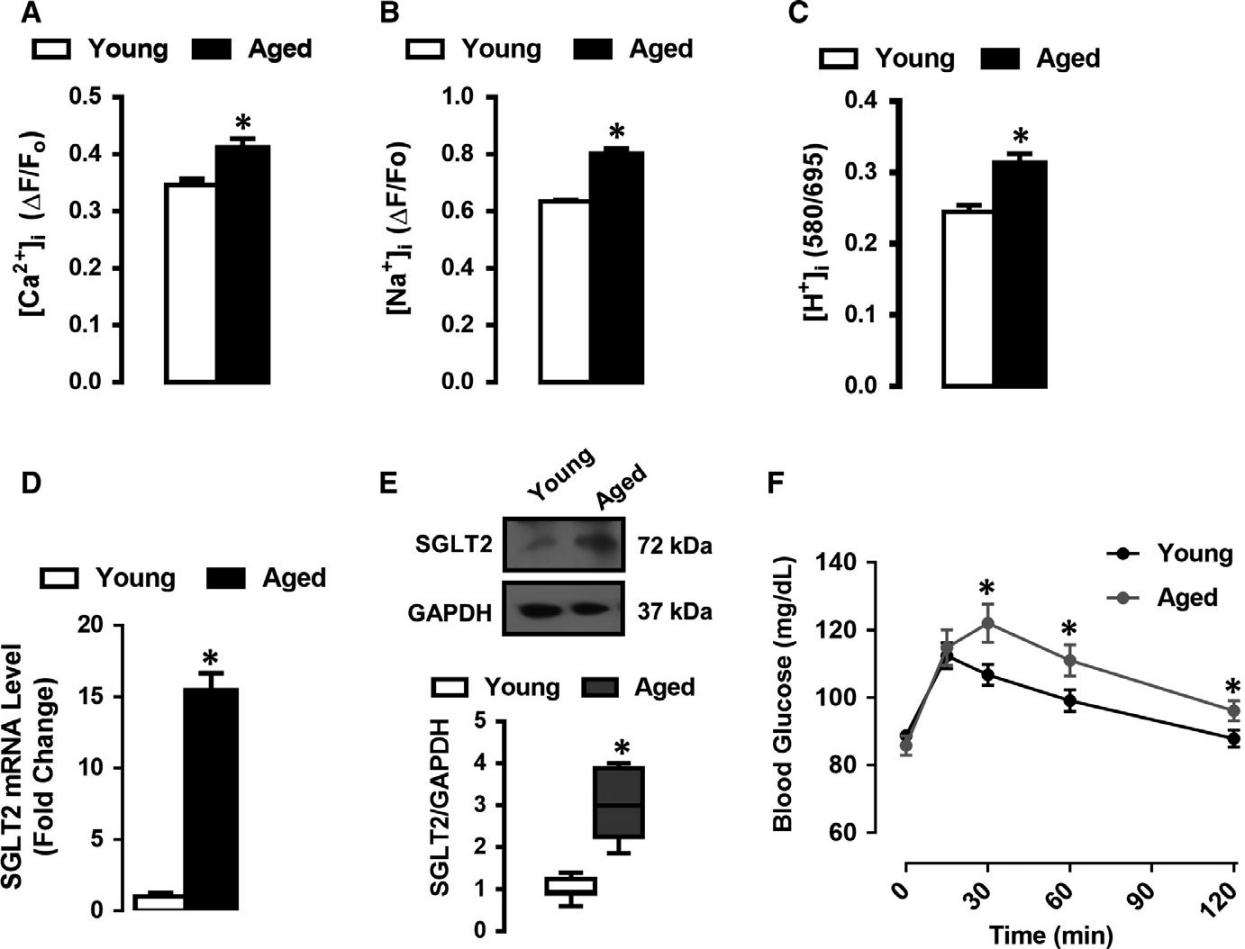
Sodium/Glucose co‐transporter 2 (SGLT2) up‐regulation increases resting levels of cytosolic ions in the aged left ventricular cardiomyocytes. The histogram presents Mean ± SEM values of resting cytosolic ions shown in Panel A, cytosolic Ca^2+^ ([Ca^2+^]_i_), Panel B, cytosolic Na^+^ ([Na^+^]_i_) and Panel C, cytosolic H^+^ ([H^+^]_i_), detected in the cells loaded with either Ca^2+^‐sensitive Fura2‐AM, Na^+^‐sensitive SBFI fluorescent dye or H^+^‐sensitive 5‐µmol/L SNARF‐1 in young and aged cardiomyocytes. Cardiomyocytes were isolated from 5‐6 rats/groups. Panel D, The mRNA levels of SGLT2 in isolated cardiomyocytes from young and old animals. Panel E, Western blot analysis of SGLT2 protein, GAPDH was used as the housekeeping gene. Panel F, The oral glucose tolerance test level, OGTT, of the aged rats compared with those of young's (n = 10 per group). The statistical significance level was ^*^ *P* < 0.05 vs. young group, analysed by the unpaired two‐sided Student's *t* test

Next, we tested the resting levels of cytosolic Ca^2+^ ([Ca^2+^]_i_), measured by fluorescence microscopy by Fura‐2 AM. As shown in Figure 4 panel A, cytosolic Ca^2^ was found to be significantly higher in aged cardiomyocytes compared with the younger cardiomyocytes. Moreover, we observed an increase in basal levels of cytosolic free Na^+^ ([Na^+^]_i_) and H^+^ ([H^+^]_i_), measured as SNARF‐1 intensity changes, in the aged cardiomyocytes, Figure 4 panels B‐C.

We previously have shown in a rat model of insulin‐resistance (MetS) that ionic balance was disrupted in an SGLT2 dependent manner.^8^ Therefore, we assessed whether age‐related abnormalities in ionic balance were related to the deregulated expression of SGLT2. We assessed SGLT2 mRNA and protein expression in young and aged cardiomyocytes. As shown in Figure 4 panels D,E, the mRNA and protein levels of SGLT2 were significantly higher in aged cardiomyocytes.

To validate the possible correlation with SGLT2 activation and insulin resistance in left ventricular aged cardiomyocytes, we performed an oral glucose tolerance test (OGTT) in aged and young rats. As shown in Figure 4 panel F, the peak blood glucose levels measured at 30, 60 and 120 minutes were significantly higher in the aged rats compared with corresponding young rats. The HOMO‐IR index was also high in the aged rats compared with the young rats (data not shown).

### 3.5 **Inhibition of SGLT2 rescues phosphorylation of PLB and suppress age‐associated mitochondrial Ca^2^** ^+^ **, [Ca^2+^]_Mit_ load in ventricular cardiomyocytes**

**FIGURE 5:**
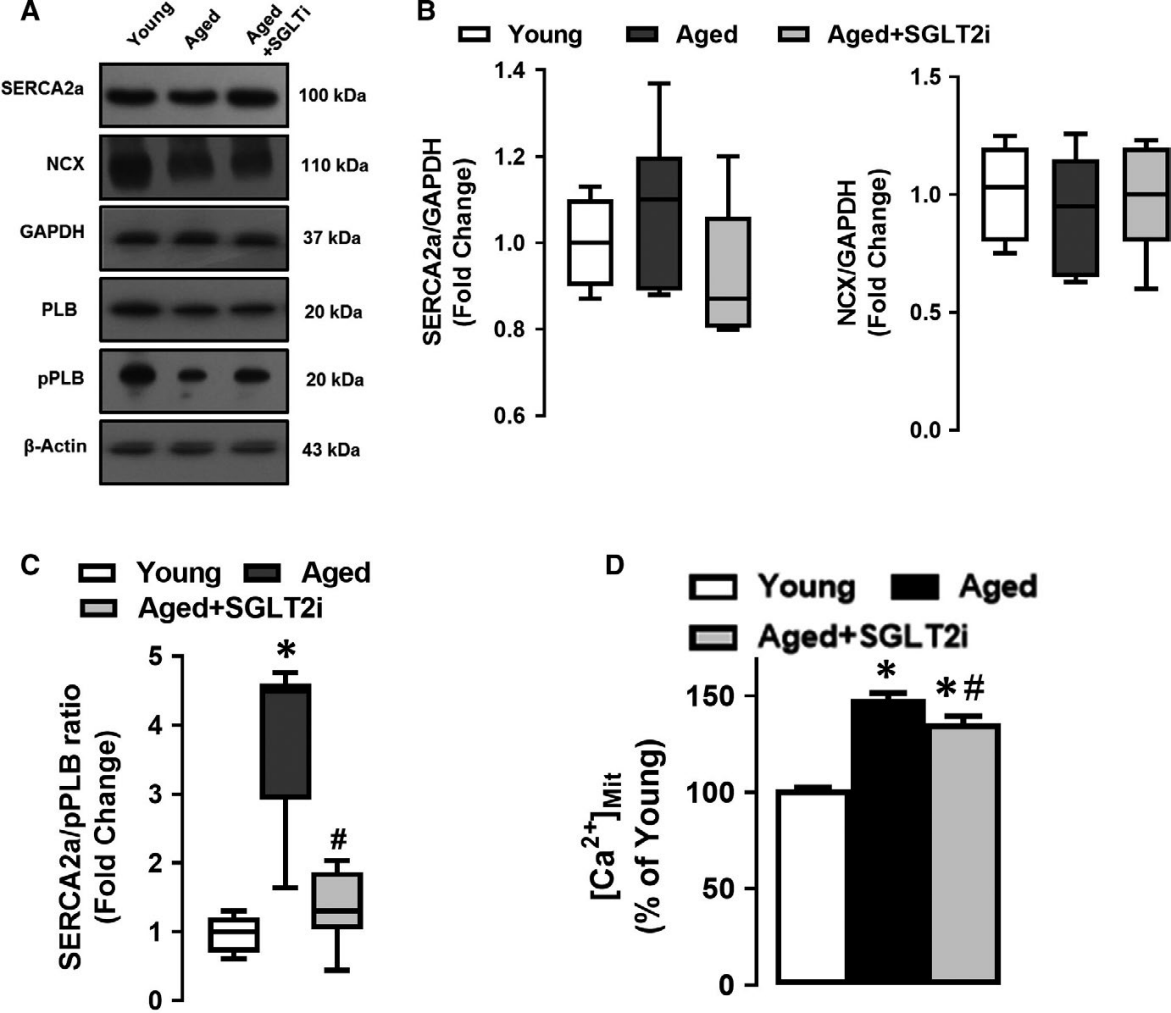
Inhibition of SGLT2 rescues phosphorylation of PLB and reduces mitochondrial Ca^2+^ load in the aged cardiomyocytes. Panel A, Western blot analysis of the lysate derived from young, aged and aged + SGLT inhibited (SGLT2i) cardiomyocytes for protein expression of sarcoplasmic reticulum Ca^2+^‐ATPase (SERCA2a), sodium/calcium‐exchanger (NCX), GAPDH; total phospholamban (PLB) and phosphorylated (pPLB) and Actin. Panels B‐C, Histogram presents fold change in SERCA2, NCX and pPLB levels of the younger group. Panel D, Histogram depicts the level of mitochondrial Ca^2+^ ([Ca^2+^]_Mit_) in Fluo4‐AM loaded (4‐µmol/L for 45‐min) young, aged and aged + SGLT inhibited (SGLT2i) cardiomyocytes. All data are presented as Mean ± SEM, cardiomyocytes were isolated from 5‐6 rats/groups. Statistical significance *P < 0.05 vs. young group, ^#^ *P* < 0.05 vs. aged group, analysed by the unpaired two‐sided Student's *t* test

Next, we assessed whether age‐related defects in Ca^2+^ homeostasis were driven by SGLT2 activation. We monitored the effect of SGLT2 inhibition (incubation with dapagliflozin; 100 nmol/L for 4‐5 hours at 37°C) on critical proteins involved in SR function. For these studies, we determined the expression level of SR Ca^2+^ ATPase (SERCA2a), Na^+^/Ca^2+^‐exchanger (NCX), PLB and phospho‐PLB (pPLB) in young and aged rats in the absence and presence of SGLT2 inhibitor. As shown in Figure 5 panels A,B, there was no significant change in SERCA2a and NCX protein levels between young, aged and SGLT2 inhibited aged cardiomyocytes. Notably, Ca^2+^ handling defects in aged cardiomyocytes were consistent with the down‐regulation of phosphorylated PLB (pPLB), a protein responsible for SERCA function, which was markedly recovered by inhibition of SGLT2, Figure 5 panels A and C. Also, SGLT2 inhibition reduced mitochondrial Ca^2+^ ([Ca^2+^]_Mit_) load in aged cardiomyocytes (Figure 5 panel D). Taken together, these data highlight an important role for SGLT2 in disrupting Ca^2+^ homeostasis at the level of SR and mitochondria in the aged cardiac myocytes; inhibition of SGLT2 can restore the SR Ca^2+^ re‐uptake and prevent Ca^2+^ build up in mitochondria of the aged cardiomyocytes.

### 3.6 Inhibition of SGLT2 suppress NaK/NCX activity and restores [Ca^2+^]_i_‐homeostasis in aged cardiomyocytes

**FIGURE 6:**
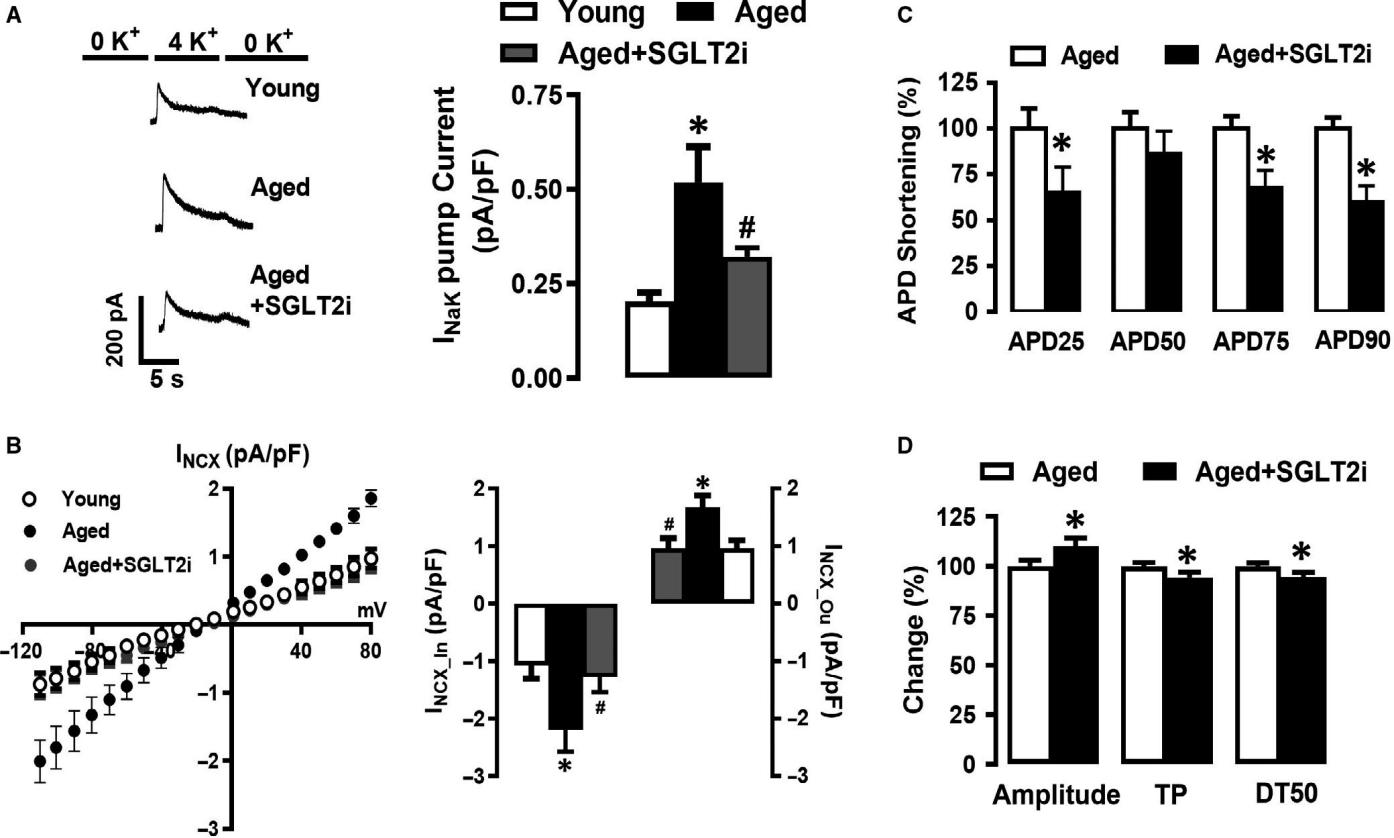
Inhibition of SGLT2 suppress deregulated electrical activities of NaK and NCX and restore [Ca^2+^]_i_‐homeostasis in aged left ventricular cardiomyocytes. Panel A, Na^+^/K^+^‐pump currents (I_NaK_) traces (left panel) in young, aged and aged + SGLT2 inhibited (SGLT2i) cardiomyocytes (treated with dapagliflozin; 100‐nmol/L D185360; for 4‐5 h), Right panel: Histogram presents Mean ± SEM values for I_NaK_ current. Panel B, Left panel, shows Na^+^/Ca^2+^‐exchanges currents (I_NCX_) (inward; I_NCXin_ and outward; I_NCXou_) and the maximum amplitudes of the currents, Right panel: Histogram presenting Mean ± SEM values for NCX activity. Panel C, The effect of SGLT2 inhibition (SGLT2i) on prolonged AP repolarization phases in aged cardiomyocytes (measured at AP_25_, AP_50_, AP_75_ and AP_90_). The histogram presents Mean ± SEM values, statistical significance *P < 0.05 vs. aged group. Panel D, SGLT2 inhibition provided important protection in the depressed Ca^2+^‐release and re‐uptake (amplitude and time course of [Ca^2+^]_i_ changes such as TP: the time to the maximum amplitude of [Ca^2+^]_i_ and RT_50_: the half time re‐uptake of [Ca^2+^]_i_; left and right panels respectively) under electrical‐field stimulation in the aged cardiomyocytes. The bar graphs present a per cent (%) change from the aged group. The statistical significance level was ^*^ *P* < 0.05 vs. aged group, analysed by unpaired two‐sided Student's *t* test

As significantly higher [Ca^2+^]_i_ and [Na^+^]_i_ were observed in aged cardiomyocytes with increased SGLT2 activity, we examined whether SGLT2 influences Na^+^/K^+^‐pump (NaK) or NCX activity. Interestingly, we observed a twofold increase in NaK current (I_NaK_) in the aged cardiomyocytes compared with corresponding young hearts, Figure 6 panel A. Furthermore, we observed a similar increase in the NCX‐currents in both forward and reversed directions (I_NCXin_ and I_NCXou_, respectively) in the aged cardiac myocytes, Figure 6, panel B. Notably, the SGLT2 inhibitor dapagliflozin (100 nmol/L) restored I_NaK_ and I_NCX_ currents, respectively, in the aged cardiomyocytes highlighting the importance of SGLT2 in the regulation of intracellular ion‐exchange currents, Figure 6 panels A,B.

Given these findings, we examined the effects of inhibition of SGLT2 on action potential (AP) prolongation in aged cardiac myocytes. As shown in Figure 6 panel C, SGLT2 inhibition significantly improved AP repolarization in the aged cardiomyocytes. Furthermore, SGLT2 inhibition improved recovery of depressed Ca^2+^‐release, indicated by a significant recovery in the amplitude of [Ca^2+^]_i_ under electrical‐field stimulation in aged cardiomyocytes (Figure 6 panel D). Collectively, these findings substantiate the involvement of SGLT in the regulation of SR and mitochondrial Ca^2+^ homeostasis during ageing.

## 4 DISCUSSION

Ageing is a major factor that predisposes cardiac myocytes to metabolic and contractile abnormalities that leads to heart failure. Lipid peroxidation, oxidative stress and mitochondrial injury are central features of insulin resistance and obesity associated with advanced age.^31^, ^32^, ^33^ Notably, mitochondrial perturbations, coupled with abnormalities in SR Ca^2+^ dysfunction have been identified as important contributors to the metabolic derangements with the ageing process in muscle cells.^34^, ^35^, ^36^ Herein, we provide new important evidence for the involvement of the sodium/glucose transporter, SGLT2 as a key regulator of cellular [Ca^2+^]_i_ homeostasis and age‐associated cardiac dysfunction. Our data demonstrate that ventricular cardiomyocytes isolated from aged‐rat hearts display mitochondrial abnormalities such as increased production of mitochondrial ROS and [Ca^2+^]_Mit_, reduced mitochondrial membrane potential (MMP) and altered expression of mitochondrial quality control proteins. The underlying mechanism for ageing‐associated increased ROS production is unclear. However, it may have resulted via an electron‐transport leak from mitochondrial complex I or complex III associated with [Ca^2+^]_i_‐overload. Furthermore, carbonylation of respiratory electron transport chain proteins resulting in monoamine oxidase activity and mPTP opening from excess mitochondrial Ca^2+^ (increased [Ca^2+^]_Mit_) may also underline the ageing‐associated increases in ROS production.^37^ Furthermore, the role of oxidative stress in exacerbating mitochondrial health in aged cardiomyocytes is supported by our MitoTempo (antioxidant) data demonstrating that MitoTempo by suppressing mitochondrial ROS production prevented the loss of mitochondrial membrane potential (MMP) and normalized dynamics protein involved in fission and fusion. This view is consistent with a report showing [Ca^2+^]_Mit_ overload disrupted normal cardiac function.^38^ Furthermore, we observed that Ca^2+^ signalling defects were not restricted to mitochondria. Our data also demonstrated a marked increase in cytosolic Ca^2+^ level ([Ca^2+^]_i,_). This observation suggests that defects in intracellular Ca^2+^ signalling up‐stream of mitochondria likely account for the observed increased [Ca^2+^]_Mit_: This view is concordant with a report suggesting that under conditions where [Ca^2+^]_i_ is high, the tendency of mitochondrial Ca^2+^‐uptake can increase by as much as 10‐ to 1,000‐fold, resulting an overload in [Ca^2+^]_Mit_.^6^

In addition to [Ca^2+^]_i,_ we also observed a marked increase in basal [Na^+^]_i_ in aged cardiomyocytes. The increased basal [Ca^2+^]_i_, in this case, maybe in part because of the depressed activity of PLB or leaky‐RyR2.^39^, ^40^ Consistent with this interpretation, we and others demonstrated that both depressed phospho‐PLB and high frequency of Ca^2+^‐sparks with high gating properties can contribute to the high [Ca^2+^]_i_ in aged cardiomyocytes. Furthermore, we observed high basal levels of [Na^+^]_i_ in aged cardiomyocytes with higher NCX activities detected both in forward and reverse directions. If NCX is operationally higher in reverse mode, this would permit high Na^+^–influx into cells, whereas in the forward mode would provide a major Ca^2+^–efflux mechanism.^25^, ^39^ Alternatively, the observed increased [Na^+^]_i_ could be because of the activation of SGLT2 in aged cardiomyocytes as we observed in our previous study in insulin‐resistant MetS rats.^8^ Herein, we show that SGLT2 was highly activated in ventricular cardiomyocytes of aged rats. We further demonstrate that SGLT2 contributes to dearrangements in [Ca^2+^]_i_ and [Na^+^]_i_ in aged cardiomyocytes, as the SGLT2 inhibitor (dapagliflozin) normalized the age‐induced defects on intracellular [Ca^2+^]_i_ and [Na^+^]_i_. As SGLT2 inhibition could confer significant protection against age‐associated defects in intracellular ion levels, we believe that SGLT2, when activated during ageing, contributes to aberrant alterations in [Ca^2+^]_i_ and [Na^+^]_i_ in cardiac myocytes. Our present data are in line with recent studies highlighting a role for SGLT2 inhibitors, on cardiometabolic, bioenergetics as well as the cardiac structure and NHE‐1 activity.^12^, ^13^, ^14^ Previous, studies have shown that an increase in myocardial Na^+^ level and Ca^2+^ level are a potential early drivers of death from cardiovascular diseases including heart failure, via induction of increased oxidative stress.^15^ Furthermore, it has also been shown that increased intracellular Na^+^ can trigger mitochondrial ROS production in the diabetic heart through activation of SGLT2.^14^, ^16^ Moreover, our present data demonstrating elevated levels of Na^+^, Ca^2+^ and H^+^ in aged cardiomyocytes is concordant with previous studies which demonstrated normalization of the activated NHE‐1 and increased intracellular Na^+^ in cardiomyocytes under pathological conditions.^12^, ^13^, ^16^, ^17^

Our data highlight that de‐regulated SGLT2 activity in cardiomyocytes promotes defective Ca^2+^‐handling that impairs contractile function with advanced age. This view is supported by recent clinical studies, demonstrating the use of metformin along with SGLT2 inhibition, in elderly patients.^41^, ^42^ Further, several clinical trials have demonstrated the beneficial effects of SGLT2 inhibitors on reducing cardiac morbidity risk of heart failure in the aged population.^10^, ^11^, ^12^, ^43^

Hence, our data suggest that deregulated SGLT2 during ageing disrupts mitochondrial function and cardiac contractility. Those effects seem to be associated with a mechanism that impinges upon deregulated [Ca^2+^]_i_ homeostasis. Our studies support the notion that interventions that modulate SGLT2 activity may provide benefits in maintaining [Ca^2+^]_i_ levels and cardiac function with advanced age. More importantly, our data demonstrate for the first time that ageing increases SGLT2 expression which negatively impacts mitochondrial/SR Ca^2+^‐homeostasis and cardiac function. In aggregate, our data provide novel evidence that SGLT2 is an important regulator of cardiac dysfunction with advanced age. Our studies further supported the notion that interventions that modulate SGLT2 activity may provide benefits in maintaining [Ca^2+^]_i_ levels and cardiac function with advanced age. Hence, targeted therapy for SGL2 inhibition may prove beneficial in protection against age‐associated contractile dysfunction.

## CONFLICT OF INTEREST

No conflicts of interest, financial or otherwise, are declared by the authors.

## AUTHOR CONTRIBUTION

**Yusuf Olgar:** Formal analysis (equal); Investigation (equal). **Erkan Tuncay:** Data curation (equal); Investigation (equal); Methodology (equal). **Sinan Degirmenci:** Investigation (equal). **Deniz Billur:** Investigation (equal). **Rimpy Dhingra:** Visualization (equal). **Lorrie Kirshenbaum:** Supervision (equal); Writing‐review & editing (equal). **Belma Turan:** Conceptualization (equal); Funding acquisition (equal); Project administration (equal); Writing‐original draft (equal). 

## Supporting information

App S1

## Data Availability

The data used to support the findings of this study are available from the corresponding author upon reasonable request.
