# Supplementary material for: Ageing‐associated increase in SGLT2 disrupts mitochondrial/sarcoplasmic reticulum Ca2+ homeostasis and promotes cardiac dysfunction
Source: J Cell Mol Med. 2020 Jul 11;24(15):8567–78. doi: 10.1111/jcmm.15483 (PMC7412693; doi:10.1111/jcmm.15483)
Supplement: Supplementary file 1 — App S1 [file JCMM-24-8567-s001.doc]

# **SUPPLEMENTARY DOCUMENTS**

# **METHODS and MATERIALS**

**Measurements of mitochondrial Ca^2+^ level and mitochondrial membrane potential (MMP)** were performed simultaneously by using confocal microscopy. As described previously in the Method section, left ventricular cardiomyocytes isolated from rat heart are incubated with 20-nM TMRM and 4-µM Fluo-4 AM for 45-min at room temperature in a solution contains (in mmol/L) 156 NaCl, 3 KCl, 2 MgSO_4_, 1.25 KH_2_PO_4_, 10 D-glucose, 2 CaCl_2_ and 10 HEPES, at pH 7.35. After loading the cells with TMRM and Fluo-4, they were washed with Ca^2+^ free HBSS twice to remove excess dye and then incubated with another solution contains (in mmol/L); NaCl 6, KCl 130, MgCl_2_ 7.8, KH_2_PO4 1, CaCl_2_ 0.4, EGTA 2, EDTA 10, malate 2, glutamate 2, ADP 2 and HEPES 20, at pH 7.1 solution including digitonin (25 mg/mL), 200-nM TMRM, 25-µg/ml digitonin and thapsigargin (10-µM) for 10-min at room temperature (1). Cardiomyocytes are placed into chambers for fluorescence imaging with a confocal microscope. TMRM and Fluo4 are excited at 543-nm and 473-nm and emission collected at 515±15-nm and 575±15-nm, respectively. The final Ca^2+^ level in the chamber was calculated using Maxchelator software.

**Measurements of electrophysiological parameters in isolated cardiomyocytes**

An Axopatch 200B amplifier with the software of pCLAMP 10.0 and Digidata 1440A analog-to-digital converter (Axon Instruments) was used to determine electrophysiological parameters of freshly isolated left ventricular cardiomyocytes. Electrophysiological traces were sampled and digitized at 5 kHz and filtered at 3 kHz with the software Digidata. Liquid-junction-potential in the amplifier was compensated before establishing the gigaseal and no leak or capacitance subtractions were performed during the current and voltage recordings. A borosilicate glass capillary tube with 2-3 MΩ to avoid internal dialysis was used during the electrophysiological parameter’s measurement.

**The TTX-sensitive Na^+^-channel currents (I_Na_)** were determined at room temperature as by using the whole-cell patch-clamping, described, previously (22±1°C) (2,3). Borosilicate glass capillary tubes were used for electrode preparation. Pulled pipettes were filled with a solution containing (as mmol/L) Cs-aspartate 120, KCl 20, NaCl 10, HEPES 10, MgATP 5, and pH adjusted to 7.2 with CsOH. As can be seen in SF2, a pre-pulse protocol (holding potential at -80 mV) was used to record these currents and calculated as a difference between negative peak and the current obtained at the end of the pulse. Every current level (pA) for every potential value was divided by its membrane capacitance (pF) for the presentation of the current density. The current–voltage relation of the channels, as well as the inactivation and reactivation properties of the channels, were calculated from on-line current recordings. A bathing low Na^+^-HEPES solution containing (in mmol/L) NaCl 40, N-methyl-D-glucamine 77, CsCl 20, CaCl_2_ 1.8, MgCl_2_ 1.8, CdCl_2_ 0.2, glucose 10, HEPES 10 and pH at 7.4 with HCl was used for the current recordings.

**The voltage-dependent L-type Ca^2+^-channel currents (I_CaL_)** were recorded and calculated at room temperature, as described previously, as well (1,2). The composition of the bathing solution containing (in mmol/L) NaCl 117, CsCl 20, MgCl_2_ 1.7, CaCl_2_ 1.8, HEPES 10 and glucose 10 was used. Voltage clamp protocol contained a pre-pulse from −70 to −55 mV (for inactivating the Na^+^ currents), followed by 300-ms depolarizing voltage steps between −60 and +80 mV.

**FIGURE LEGENDS**

**SF.1.** **Determination of mitochondrial Ca^2+^ level and mitochondrial membrane potential (MMP), simultaneously, by using confocal microscopy.**

As described previously in the method section, cardiomyocytes isolated from rat heart were incubated with 20-nM TMRM and 4-µM Fluo-4 AM for 45-min at room temperature. TMRM and Fluo-4 loaded cells were excited at 543 nm, 473 nm and emission collected at 515±15 nm and 575±15 nm, respectively. Following the application of CaCl_2_ (128-nM), the final Ca^2+^ level in the chamber was calculated by using Maxchelator software, which was detecting the increase in the intensities of Fluo-4 fluorescence and the decrease in the intensities of TMRM fluorescence (right panel).

**SF.2.** **The voltage-dependent TTX-sensitive Na^+^-channel currents and voltage-dependent L-type Ca^2+^channel** **currents measured in cardiomyocytes isolated from aged rat heart.** **Panel A**: The current-voltage relations (current-voltage, I-V, characteristics) of voltage-dependent TTX-sensitive Na^+^-channels **(left panel)** with a comparison between the young and aged group. The calculated maximum currents of Na^+^-channels, I_Na_ (at -40 mV) and their measurement protocols are given in the lower parts of I-V graphs. The reactivation (middle) and inactivation **(right panel)** characteristics of those channels are given, respectively. **Panel B**: The current-voltage relations (current-voltage, I-V, characteristics) of voltage-dependent L-type Ca^2+^**-**channels for these two groups. The calculated maximum currents of Ca^2+^-channels, I_CaL_ (at 0 mV) and their measurement protocols are given in the lower parts of I-V graphs **(left panel)** The reactivation **(middle panel)** and inactivation **(right panel)** characteristics of those channels are given, respectively. The values are presented as Mean (±SEM). Statistical significance level ^*^p<0.05 *vs.* the young-group, analyzed by unpaired two-sided Student's t-test.

**REFERENCES**

1. McKenzie M, Lim SC and Duchen MR (2017) Simultaneous Measurement of Mitochondrial Calcium and Mitochondrial Membrane Potential in Live Cells by Fluorescent Microscopy. J Vis Exp. doi: 10.3791/55166.

2. Bilginoglu A, Kandilci HB, Turan B. Intracellular levels of Na(+) and TTX-sensitive Na(+) channel current in diabetic rat ventricular cardiomyocytes. *Cardiovasc Toxicol* 2013;**13**:138-147.

3. Turan B, Desilets M, Acan LN, Hotomaroglu O, Vannier C, Vassort G. Oxidative effects of selenite on rat ventricular contractility and Ca movements. *Cardiovasc Res* 1996;**32**:351-361.


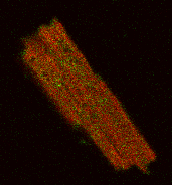

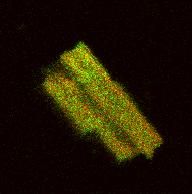


**+CaCl_2_**


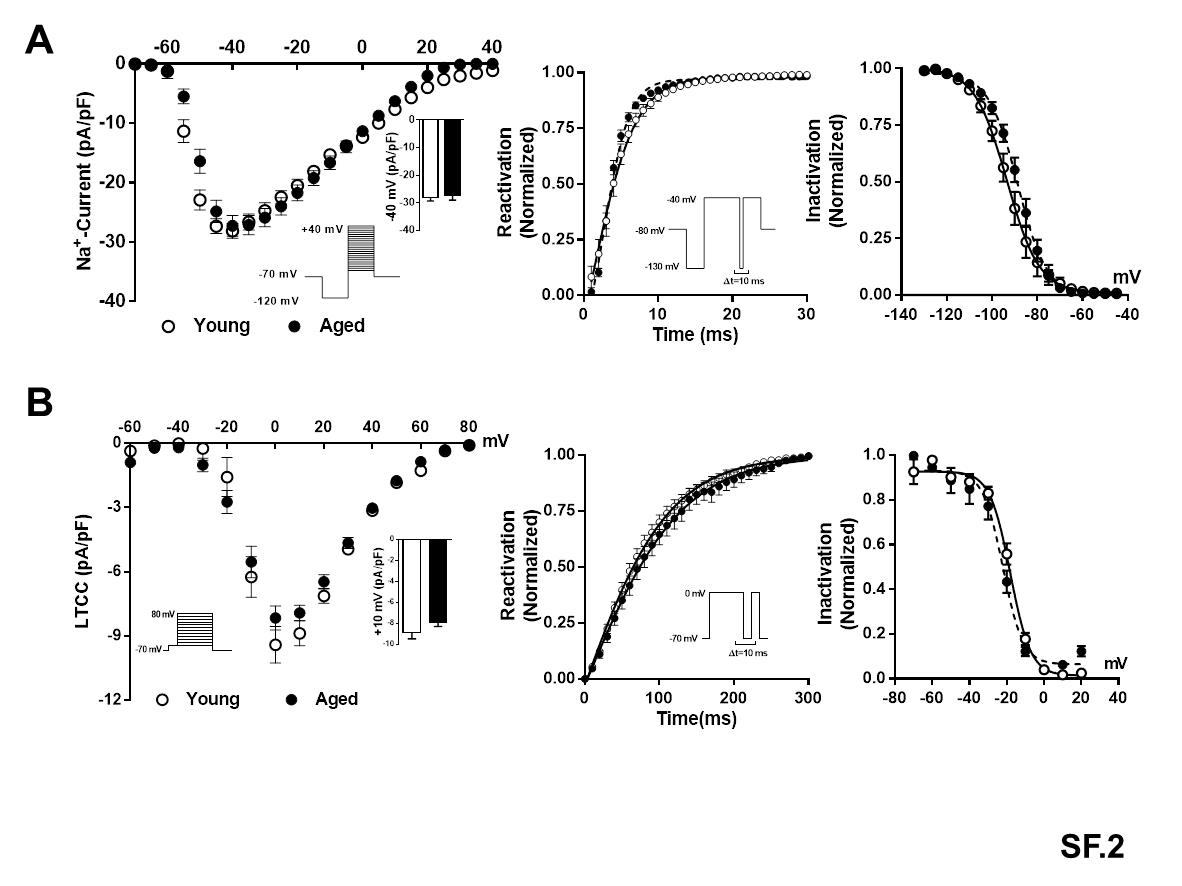
 **SF.1**

**SF.2**
